# Modeling Core Metabolism in Cancer Cells: Surveying the Topology Underlying the Warburg Effect

**DOI:** 10.1371/journal.pone.0012383

**Published:** 2010-08-25

**Authors:** Osbaldo Resendis-Antonio, Alberto Checa, Sergio Encarnación

**Affiliations:** Centro de Ciencias Genomicas-Universidad Nacional Autónoma de México, Cuernavaca, México; Memorial Sloan-Kettering Cancer Center, United States of America

## Abstract

**Background:**

Alterations on glucose consumption and biosynthetic activity of amino acids, lipids and nucleotides are metabolic changes for sustaining cell proliferation in cancer cells. Irrevocable evidence of this fact is the Warburg effect which establishes that cancer cells prefers glycolysis over oxidative phosphorylation to generate ATP. Regulatory action over metabolic enzymes has opened a new window for designing more effective anti-cancer treatments. This enterprise is not trivial and the development of computational models that contribute to identifying potential enzymes for breaking the robustness of cancer cells is a priority.

**Methodology/Principal Findings:**

This work presents a constraint-base modeling of the most experimentally studied metabolic pathways supporting cancer cells: *glycolysis*, *TCA cycle*, *pentose phosphate*, *glutaminolysis and oxidative phosphorylation*. To evaluate its predictive capacities, a growth kinetics study for *Hela* cell lines was accomplished and qualitatively compared with *in silico* predictions. Furthermore, based on pure computational criteria, we concluded that a set of enzymes (such as *lactate dehydrogenase* and *pyruvate dehydrogenase*) perform a pivotal role in cancer cell growth, findings supported by an experimental counterpart.

**Conclusions/Significance:**

Alterations on metabolic activity are crucial to initiate and sustain cancer phenotype. In this work, we analyzed the phenotype capacities emerged from a constructed metabolic network conformed by the most experimentally studied pathways sustaining cancer cell growth. Remarkably, *in silico* model was able to resemble the physiological conditions in cancer cells and successfully identified some enzymes currently studied by its therapeutic effect. Overall, we supplied evidence that constraint-based modeling constitutes a promising computational platform to: 1) integrate high throughput technology and establish a crosstalk between experimental validation and *in silico* prediction in cancer cell phenotype; 2) explore the fundamental metabolic mechanism that confers robustness in cancer; and 3) suggest new metabolic targets for anticancer treatments. All these issues being central to explore cancer cell metabolism from a systems biology perspective.

## Introduction

In recent years we have witnessed significative advances for identifying and understanding the role that individual genes have in genesis, development and progression on cancer [1]. However, despite significant advances in genomic sciences in identifying oncogenes and tumor suppressors, a systemic explanation of how these genes deregulate the normal function of genetic circuits and how its control may be used to design effective drugs against cancer still remains a great challenge in systems biology [2], [3], [4], [5], [6].

In conjunction with this molecular view of cancer, detailed studies monitoring the metabolic alterations in cells are a promising avenue for understanding and controlling cell proliferation in cancer cells [2], [7], [8]. For instance, researchers have extensively studied the *p53* tumor suppressor's ability to trigger *DNA repair*, cell cycle arrest and apoptosis, but recently *p53*'s capacity to influence mitochondrial respiration and energy metabolism have been elucidated [9], [10]. Similarly, enhanced effect on glycolysis, lactate (*lac*) production and control of fatty acids oxidation originated by *Hypoxia inducible factors (HIF)* and *LKB1* tumor suppressor are clear examples linking genes expression, metabolism and cancer phenotype [3].

In this contextual scheme, development of computational procedures capable of surveying the physiological responses on cancer cells in terms of its metabolic topology and genetic information constitutes an attractive strategy for understanding, characterizing, designing and improving effectiveness of cancer drugs [11]. In this work we present a constraint-based analysis of a metabolic network integrated by a core of metabolic pathways participating in cancer cell growth: *glycolysis*, *TCA cycle*, *pentose phosphate pathways (PPP) and oxidative phosphorylation*. Constraint-based modeling has proven to be a successful paradigm in systems biology for describing and exploring the phenotype capacities for a variety of organisms based on its particular genome sequences and metabolic topology [11], [12], [13], [14], [15].

This paper's central aim is twofold: 1) the construction of a model simulating metabolisms that serves as a computational framework auxiliary to describe and understand physiological behavior in cancer cells; and 2) the identification of potential metabolic targets to induce a reduced phenotype on cancer cell growth. To qualitatively assess the *in silico* results obtained from our metabolic reconstruction with those experimentally observed, we accomplished a study of growth kinetics for *Hela* cell line. Furthermore, based on computational criteria we identified some enzymes with a relevant influence on cell growth and compared them with those considered as potential therapeutic targets in the literature.

Overall, we supply evidence that constraint-based modeling can be used as a platform for unraveling the biochemical mechanism underlying cancer cell growth and potentially contribute toward designing strategies for clinical treatments in cancer.

## Results

### Core Metabolism in cancer cells

Ever since the pioneering observation that aerobic glycolysis in cancer [7] is preferred over *oxidative phosphorylation* as a mechanism to generate *ATP* from glucose, numerous experiments have supported and extended the significant role that metabolisms have on transformation, proliferation, angiogenesis and metastasis in cancer [16], [17], [18]. Thus, scanning human tumors with *positron emission tomography* (*PET*) [17] has verified that a high uptake rate of glucose constitutes a hallmark in cancer cells, presumably required to confer adaptive advantages when facing acidic and hypoxic environments [19].

In light of these observations, an explanation of why energy production relies on *glycolysis* instead of on the more effective pathway driven by *oxidative phosphorylation* in *mitochondria* [2], [16] requires computational models capable of taking into account not only both pathways but a robust metabolic network containing its metabolic interconnectivity.

**Figure 1:**
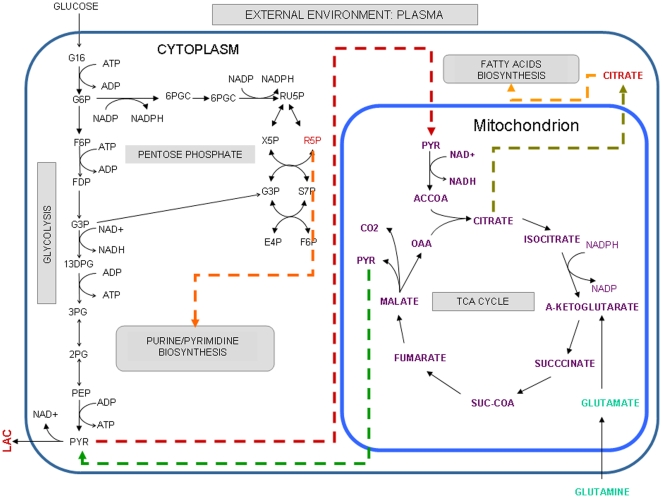
Metabolic pathways with a significant role in cancer cells. As a result of a bibliography search, we have selected those metabolic pathways that potentially can constitute a metabolic core on most cancer cells. Orange, red and green dashed lines indicate metabolites that participate in other biosynthetic pathways, metabolites that can be transported from cytoplasm to mitochondrion and metabolites that can be transported from mitochondrion to cytoplasm, respectively. Compartment information has been denoted by external environment [e], cytoplasm [c] and mitochondria [m]. The set of reactions that integrates this reconstruction are listed in Table S1.

Keeping in mind this systemic view, we constructed a metabolic network with those metabolic pathways that have a pivotal role in cancer cell growth: *glycolysis*, *TCA cycle*, *pentose phosphate*, *glutaminolysis and oxidative phosphorylation* [3], [16]. According to reconstruction protocols, our network was based on published knowledge about metabolism in cancer cells, basic thermodynamics and compartmentalization information associated with each metabolic reaction inside the cell, see Table S1. Thus, for example, studies on *C^13^ NMR* spectroscopy have demonstrated that glutaminolysis constitutes an active metabolic pathway in human glioblastoma cell lines [8], and consequently, a demand reaction of *α-ketoglutarate* representing an intermediary compound along the conversion of glutamine to lactate was included in the reconstruction. In addition, the reconstruction was complemented by transport reactions to resemble the physiological conditions prevailing in cancer cells, particularly those associated with *glucose* consumption, *lactate* production and *hypoxia* conditions, *see Table S1*. Overall, our reconstruction integrates 66 metabolites participating in 80 metabolic reactions representing *glycolysis*, *pentose phosphate*, *TCA cycle*, *oxidative phosphorylation* and *glutaminolysis*, as well as transport reactions of essential metabolites for cellular proliferation, specifically *oxygen*, *hydrogen*, *carbon dioxide* and *water*, see Table S1 in supplementary material. *Figure 1* depicts the metabolic network used in this study. Mathematical representation of this set of reactions, through the stoichiometric matrix, constitutes our central platform for exploring and estimating the metabolic capacities potentially driving cancer cells [3], [16].

### Dynamic Constraint-based Modeling and its experimental assessment

Experimental assessment of the results and hypothesis inferred from computational modeling is needed to ensure a high-quality metabolic reconstruction with a real scope for explaining and predicting cell behavior. Given that self-sufficiency in growth signals and mechanisms for evading apoptosis [20] in cancer cells contribute to uncontrolled cell proliferation, the feasibility of our model to simulate cancer cell growth constituted a principal issue to evaluate. Therefore, dynamic constraint-based modeling was applied to the metabolic reconstruction depicted in *Figure 1*. According to this formalism, growth rate is calculated by assuming the existence of a characteristic time scale, at which a steady state condition for metabolite concentrations is a plausible assumption. Thus, hypothesizing that physiological growth rate at each time scale obeys optimization principles, linear programming was applied to identify the metabolic flux profile that maximized a function associated with growth rate [21], see methods section.

Malignant progression requires proper metabolic cell machinery in order to supply the energy and biosynthetic demand required for cancer cell growth. To quantify the cancer cell growth in terms of its metabolic networks and to link the topology of the reconstruction with the cancer cell physiology, we proceeded to construct an objective function that mathematically represents the metabolic demands required for successful cell growth [11], [13], [22], [23].

The proper selection of an objective function is crucial for reducing the steady-state stoichiometrically feasible solution to an optimal solution space [22], [24]. In this work the objective function was created by taking into account the expected metabolites supporting cancer cell proliferation [25]. Thus, based on a review of literature and considering the set of metabolites integrating our reconstruction, we suggest an objective function consisting of *lactate (lac)*, *ATP*, *ribose 5-phosphate (r5p)*, *oxaloacetate (oaa)* and *citrate(cit)* production, having been selected according to their fundamental roles as 1) precursors required for energy production, 2) precursors of amino acids and nucleotides and 3) intermediates in maintaining glycolysis and the reductive power required for biosynthesis of other cellular compounds [25], [26]:
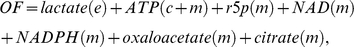
where *c*, *e* and *m* denote the compartments utilized in the reconstruction (*cytoplasm*, *external environment* and *mitochondria* respectively).

In addition, proper computational representation of environmental conditions is essential to obtain reliable results and interpretations from *in silico* procedures [24]. Hence, *sink* and *demand* reactions were included to define proper metabolic boundaries to mimic the physiological conditions prevailing around cancer cells, see Table S1. Through sink reactions (which serve to introduce those metabolites that are produced or consumed by nonmetabolic cellular processes), we represent *NADH*, *NAD*, *CO2*, *biphosphate*, *hydrogen*, *water*, *carbon dioxide*, *coenzyme A*, *FAD* and *FADH2*. In turn, through demand reactions (which are unbalanced reactions that permit the accumulation of a compound otherwise not allowed in steady-state models because of mass-balancing requirements), we were able to include a source of *ACCOA*, *ADP* and *oxygen*.

Furthermore, plasma, an abundant source of glucose and glutamine in cancer cells, was represented by two demand reactions in the reconstruction, see Figure 1 and Table S1. In order to simulate glucose consumption, a simple transport of glucose was included in the metabolic reconstruction, while consumption on glutamine was represented through an external source of *2-oxoglutarate*, one of the intermediary products of the glutaminolysis pathway in cancer cells, see Figure 1 [8].

Finally, consistent with hypoxia conditions governing cancer cell environment, all the simulations were constrained to low rates of oxygen uptake [27], see details in Table S1.

### Constraint-base modeling assessment : Evaluating Objective Function

**Figure 2:**
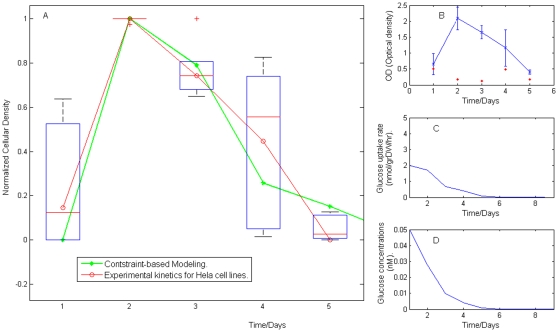
Temporal profile of kinetic cell growth. (A) Comparative analysis between the growth rate obtained experimentally and *in silico*. (B) Average and standard deviation obtained in the kinetics measurements for *Hela* cell lines. As described in methods, growth rate was monitored every 24 hours for five days, and six replicates were obtained for each absorbance measurement. Statistical properties characterizing the kinetic growth on *Hela* cell lines are shown in *Figure 2(B)*, while the temporal behavior of glucose uptake rate and external concentration predicted by *in silico* procedures are depicted in *(C)* and *(D)*, respectively. Coefficients of variation obtained at each measurement are reported by the red points in (B).

To evaluate the physiological significance of the proposed objective function, we decided to explore the extent to which growth rate derived from dynamic constraint-based modeling coincided with that obtained from a kinetic growth study of *Hela* cell lines. Therefore, the *in silico* temporal profile of growth rate was calculated by defining an initial cellular density, an initial available glucose concentration and a proper time scale for assuming steady-state condition, see methods section. Meanwhile, *Hela* cancer cell lines were cultivated in solution and a growth kinetic study was accomplished. As described in the methods section, experimental measurements of cellular density on *Hela* cells were made with six replicates for estimated experimental reproducibility and by monitoring the process every 24 hours for five days, see also Figure 2 (B).

The contribution of metabolic entities in the objective function has been assumed to carry equal weight on growth rate, in such a way that instead of using a quantitative criteria to evaluate the crosstalk between experiment and modeling, a qualitative procedure based on the normalization of cellular density profile was implemented. Thus, proceeding as described in the methods section, we found that our modeling was capable of obtaining a normalized temporal growth profile comparable with that associated with *Hela* cell lines, see Figure 2.

In light of this result, we postulate that the objective function associated with the metabolic reconstruction depicted in *Figure 1* is potentially able to elucidate the metabolic flux activity required for supplying the metabolic demand for cancer cell growth. This is a crucial contribution in this study and constitutes the backbone for exploring the relationships among gene activity, metabolism and phenotype in cancer.

### *In silico* simulations

**Figure 3:**
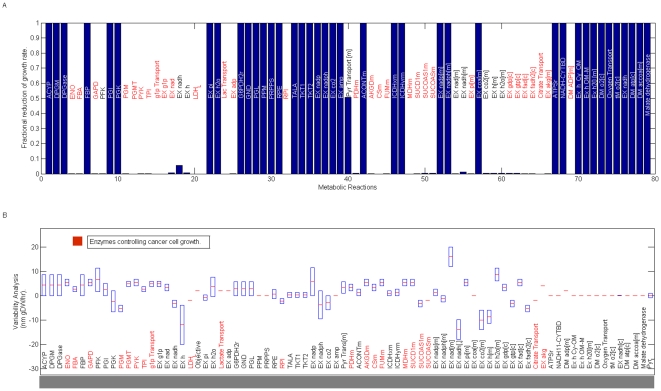
Flux Variability and Enzyme essentiality on metabolic reactions. To identify those reactions that could have a pivotal role in cancer growth rate, flux variability and enzyme essentiality analysis were accomplished over all the reactions included in the reconstruction. In panel *(A)*, the metabolic reactions whose deletion produces a significant reduction on growth rate are highlighted in red. Those reactions that ensure a low variability and high essentiality constitute 27% of the complete metabolic reconstruction and these appear in red in panel *(B)*. Exchange and sink reactions were excluded from this analysis. Abbreviation code: *Enolase (ENO)*, *glyceraldehyde-3-phosphate dehydrogenase(GAPD)*, *phosphoglucomutase (PGMT)*, *pyruvate kinase (PYK)*, *triose-phosphate isomerase (TPI)*, *lactate dehydrogenase (LDH)*, *ribose-5-phosphate isomerase (RPI)*, *pyruvate dehydrogenase (PDHm)*, *2-oxoglutarate dehydrogenase (AKGDm)*, *cytrate synthase (CSm)*, *Fumarate hydratase (FUMm)*, *malate dehydrogenase (MDHm)*, *succinate dehydrogenase (SUCD1m)*, *succinyl-CoA synthetase (SUCOAS)*.

Computational models on biological systems have two general purposes: 1) to reproduce what is physiologically observed and understand their biological principles, and 2) to create a platform capable of predicting the cellular phenotype when metabolic alterations are induced in the system. Having verified that *in silico* phenotype qualitatively reproduces the growth rate of *Hela* cell lines, we proceeded to survey the metabolic mechanisms supporting cell proliferation through *Flux Balance Analysis* (*FBA*), an *in silico* formalism that has been useful in exploring the genotype-phenotype relation for a variety of organisms [11], [12], [13], [22], [23], [28]. Specifically, we have used our metabolic reconstruction for identifying those biochemical reactions that have a strong influence on controlling cancer cell growth, a worthy issue when one desires to identify metabolic targets with effective results in cancer treatments [6]. For this purpose, metabolic targets with a central role in cancer cell growth were identified by two constraints: *low flux variability* and *high enzymatic essentiality* for cancer cell growth. Together, these constraints constitute computational criteria for selecting those reactions that ensure a low redundancy on metabolite synthesis with a maximal effect for decreasing its phenotype. Thus, this computational criteria lead us to identify a set of target enzymes whose metabolic activity may has a direct effect on cancer cell growth, see Figure 3.

**Figure 4:**
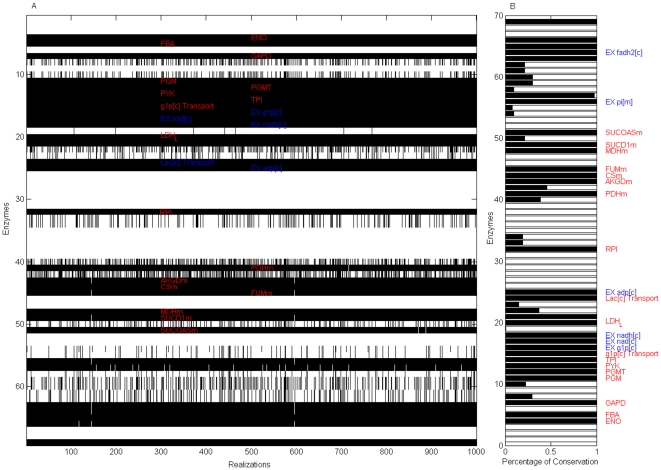
Enzymes with high essentiality and low variability that are robust to the ratio of objective function's components. Reactions with high essentiality and low variability were identified through a set of 1000 objective functions with nonequivalent ratios among function components. As panel (A) shows, reactions obeying both criteria (red on black regions) were plotted over the 1000 realizations. In each realization, those enzymes that obey the *in silico* criteria were denoted in black; all others in white. The percentage of times reactions obeyed the computational criteria are depicted in panel (B). Robust enzymes relevant to this study (excluding transporters, exchange and demand reactions) were labeled in red. EX, DM and Sink denote exchange, demand and sink reactions in the cytoplasm [c] and mitochondria [m] compartments.

The robustness of this set of target enzymes in terms of the ratios among the objective function's components was subsequently verified: We repeatedly applied the *in silico* analysis to a set of objective functions whose equimolar contributions on objective function's components were not assumed. With this in mind, 1,000 objective functions (with components selected from a random uniform distribution ranging from 0 to 1 around numerical values estimated for other organisms [22]) were reconstructed, and enzymes with *low flux variability* and *high enzymatic essentiality* were identified in each realization. Despite growth rates highly dependent on the ratios of the objective function's components, we identified a set of enzymes that in 99% of all the realizations obeyed selection criteria, *see* *Figure 4*. Among the target enzymes identified *in silico*, we determined that some participate in *glycolysis*, such as *phosphoglucomutase (PGMT)*, *enolase (ENO)*, *glyceraldehyde-3-phosphate dehydrogenase (GAPD)*, *pyruvate kinase (PYK)* and *lactate dehydrogenase (LDH)*. Consistent with this result, development of drugs mainly targeting glucose transport and phosphorylation steps in glycolytic pathways have shown to be a latent therapeutic strategy for reducing cancer phenotype [19], [27], [29].

**Figure 5:**
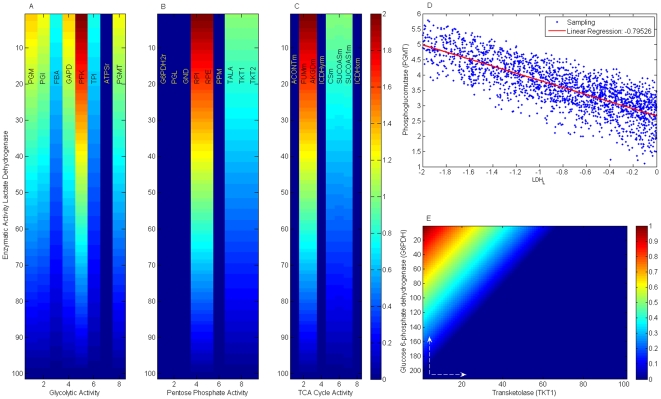
Lactate dehydrogenase and its influence on central pathways. *Lactate dehydrogenase (LDH)* has been suggested as a pivotal metabolic control on cancer cell growth with a significant role in the Warburg effect. Panels (A), (B) and (C) show the effects that variations of *LDH* activity have on some enzymes participating in *glycolysis*, *TCA cycle* and *pentose phosphate*, respectively. Metabolic activity of LDH increases from bottom to top. Panel *(D)* shows the correlation between flux activity of *LDH* and *phosphoglucomutase (PGMT)* obtained through sampling the null space of the stoichiometric matrix. Phenotype phase plane for *glucose-6-phosphate dehydrogenase* (*G6PDH*) and *transketolase* (*TKT1*), enzymes quantifying the activity of the oxidative and non-oxidative branches of pentose phosphate, is depicted in panel (E). White arrows indicate the direction at which the metabolic flux increases.

Furthermore, constraint-based modeling suggests that *lactate dehydrogenase* can be used as a metabolic control point over phenotype behavior in agreement with previous studies, *see* *Figure 5* [2], [3]. Specifically, there has been experimental evidence that inhibition of *lactate dehydrogenase* induces a decreased activity of some glycolytic enzymes and consequently reduces growth rate in cancer cells [30]. Motivated by this fact and with the purpose of further assessing our computational interpretation, we evaluated to what extent a reduction of enzymatic capacity of *lactate dehydrogenase* influences the metabolic activity on enzymes participating in *glycolysis*, *pentose phosphate* and *TCA cycle*. As *Figure 5* shows (panel A, B and C), flux balance analysis exhibits that an increment on enzyme activity for *lactate dehydrogenase* is followed by an increased metabolic activity over *glycolysis* and some enzymes participating in *TCA cycle* and *pentose phosphate pathway*. Consistent with this *in silico* observation, an increase of *lactate* production has been proposed to be a necessary condition supporting tumor cell transformation through the Warburg effect [31]. To confirm that this property is a consequence of the geometry of the flux steady-state solution space and not of the particular selections of ratios in the objective function's components, a nonbiased Monte Carlo sampling method was applied for characterizing the solution spaces [23], see methods section. As Figure 5 (D) shows, a significant correlation emerged between metabolic activity of lactate dehydrogenase (*LDH*) and the first enzyme in glycolysis: *phosphoglucomutase* (*PDGM*). A decrease of *LDH* tends to be related with a decrease on glucose metabolism through *PDGM*, hence, our *in silico* analysis suggests *LDH* as a control point in cancer cell metabolism.

On the other hand, our computational platform suggests that *pyruvate dehydrogenase (PDHm*) can perform a central role in driving cell proliferation due to its low *flux variability* and high *enzymatic essentiality* for metabolism in cancer cell growth, see Figure 4 (A). Consistent with this finding, there is evidence that metabolic inhibition of *PDHm* contributes to Warburg metabolism and enhances malignant phenotype in human neck and head squamous carcinomas [26], [32]. This observation could make sense in light of additional regulatory components integrating this metabolic puzzle. First, hypoxia condition in tumors induces the activation of *HIF (Hypoxia Inducible Factor)*, which in turn activates *pyruvate dehydrogenase kinase 1*, an enzyme that negatively regulates the catalytic activity of *PDHm*. In addition, aerobic *glycolysis* is enhanced by the fact that *HIF* induces the overproduction of enzymes participating in the glycolytic pathway and lactate production [31]. Overall, enhancement of the Warburg effect and diminishing activity of *PDHm* seems to be a metabolic response that confers selective advantage for survival and cell proliferation.

**Figure 6:**
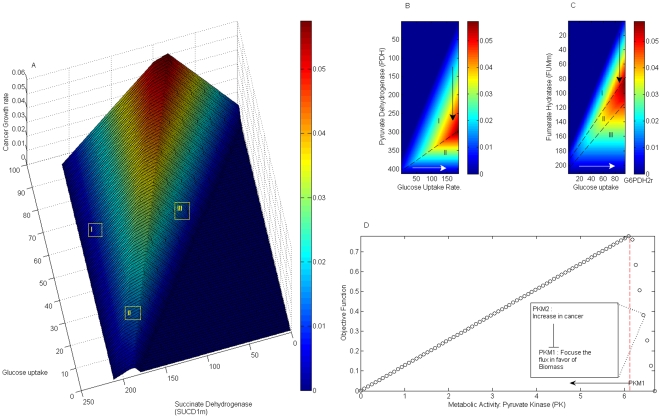
Phenotype phase plane for glycolytic and TCA cycle enzymes. Panel (A) is a three-dimensional representation of how metabolic activity of succinate dehydrogenase and glucose uptake rate influence growth. As Panels *(B) and (C)* show, *in silico* modeling leads us to identify some regions where variations on *pyruvate dehydrogenase* and *fumarate hydratase*, both associated with tumor suppressor activity, can result in different phenotypes. White lines indicate the direction at which the metabolic fluxes increase; black lines, the direction at which they decrease. The potential effect that pyruvate kinase activity can produce on cancer cell growth is depicted in *(D)*. In panel *(D)* objective function components were selected as follows: *c_ATP_* = 12.47, *c_Lactate_* = 0.13, *c_NADPH_* = 0.93, *c_R5P_* = 0.6, *c_NAD_* = 0.89, *c_OAA_* = 0.75, *c_ATP[m]_* = 17.09 and *c_Citrate_* = 0.55. The threshold flux activity is denoted by a red line.

With the purpose of surveying how growth in cancer cells may vary when changing the metabolic activities on both *PDHm* and *glucose transport*, we have accomplished *phenotypic phase plane* analysis, a computational procedure to visually explore how objective function behaves when flux variations over two independent metabolic reactions occurs [21], [23]. Remarkably, as Figure 6 (B) shows, our analysis suggests that at fix glucose uptake rate a decrease on *PDHm* enzymatic activity may improve the phenotype growth rate in cancer cell lines, arrow in region I. Despite the fact that this result is in agreement with some experimental reports, our computational model predicts the existence of a threshold on *PDHm* whose reduced activity could be beneficial to arrest cancer cell growth (region II), a result that requires posterior experimental verification.

Optimization of the objective function leads us to conclude that *glutaminolysis*, starting at *glutamine* uptake rate and ending with lactate production, is an active pathway during cancer cell growth. From a functional and biological point of view, *glutaminolysis* performs a fundamental role in replenishing *TCA cycle* and generating additional reductive power required for fatty acids biosynthesis. Furthermore, our *in silico* analysis suggests that *fumarate hydratase (FUMm)* and *succinate dehydrogenase (SUCD1m)* can be independently used as metabolic targets for regulating cell proliferation, see Figure 6A and C. Phenotype phase plane accomplished over these enzymes allows us to conclude that when activity of *FUMm* (*SUCD1M*) is reduced different regions separated by a threshold value are identified. As can be appreciated in *Figure 6* *(A)* and *(B)*, when metabolic activity on *FUMm* or *SUCD1M* is decreased, phenotype growth rate in region I is enhanced while in region III is reduced. Interestingly, phenotype behavior observed in region I is in accordance with the fact that *FUMm or SUCD1m* can participate as a tumor suppressor when its enzymatic activity is deficient [33]. Even though the model can sense the influence that the enzymatic activity of *FUMm* or *SUCD1m* has on cancer growth rate, further analysis is required to assess if *in silico* interpretation on region II and III has a biological meaning.

We highlight that in our simulations mitochondria-derived *citrate* constitutes a fundamental metabolite to be optimized for supporting cell proliferation. As a result, low citrate transport from mitochondria toward cytoplasm induces a decreased effect on *in silico* growth rate. Consistent with published findings, inhibition of *ATP citrate lyase* participating in conversion of mitochondria-derived citrate into *acetyl-coenzyme A* in cytoplasm prevents cancer cell proliferation and tumor growth due to its central role as a precursor for lipids [2], [34]. Even though inhibition of *ATP citrate lyase* and low *citrate* transport have the final effect of reducing *acetyl-coenzyme A*, a more detailed analysis should be considered in future reconstructions. Specifically, fatty acids metabolism should be included in the reconstruction in order to evaluate if cytoplasm *acetyl-coenzyme A* could be a more appropriate component than cytoplasm *citrate* to simulate computationally cancer cell growth.

The pharmaceutical design of drugs targeting the *pentose phosphate* pathway (PPP) seems to be an appealing strategy to reduce growth rate in cancer cells due to its essential role in synthesizing *ribose-5-phosphate (r5p)*, essential for biosynthesis of nucleotides and nucleic acids [35]. To quantify the contribution of PPP to cancer cell growth, we analyzed *in silico* how *transketolase (TKT1)* and *glucose-6-phosphate dehydrogenase (G6PDH)*, representing the nonoxidative and oxidative branches in *PPP* respectively, affect cell growth phenotype, see Figure 5E. As Figure 3A shows, *in silico* mutation of *TKT or G6PDH* predicts its nonessentiality in cancer cell growth, this due to the fact that non-oxidative or oxidative branches in *PPP* can both produce *ribose-5 phosphate (r5p)*. Converse to this harmless effect of simple mutations, however, *in silico* modeling suggests that simultaneous mutation is lethal for phenotype in cancer cell growth, a finding that agrees with already published reports. Thus, enzymatic down-regulation of *glucose-6-phosphate dehydrogenase (G6PDH)* and *TKT1* is required to effectively arrest growth rate for cancer cells in animals [6], [35]. Furthermore, phenotype phase plane done with *G6PDH* and *TKT1* allows us to infer not only that a simultaneous reduction of *TKT1* and *G6PDH* metabolic activity clearly predicts a drop in growth rate but also that, although both branches of *PPP* can produce *r5p*, the *oxidative* branch can be a limiting factor for growth rate in cancer: it means if *TKT1* flux is null an increment of *G6PDH* metabolic activity can produce phenotype, however the opposite situation does not occurs, *see* *Figure 5(E)*, a hypothesis that needs to be verified with more precise experimental measurements [35].

**Figure 7:**
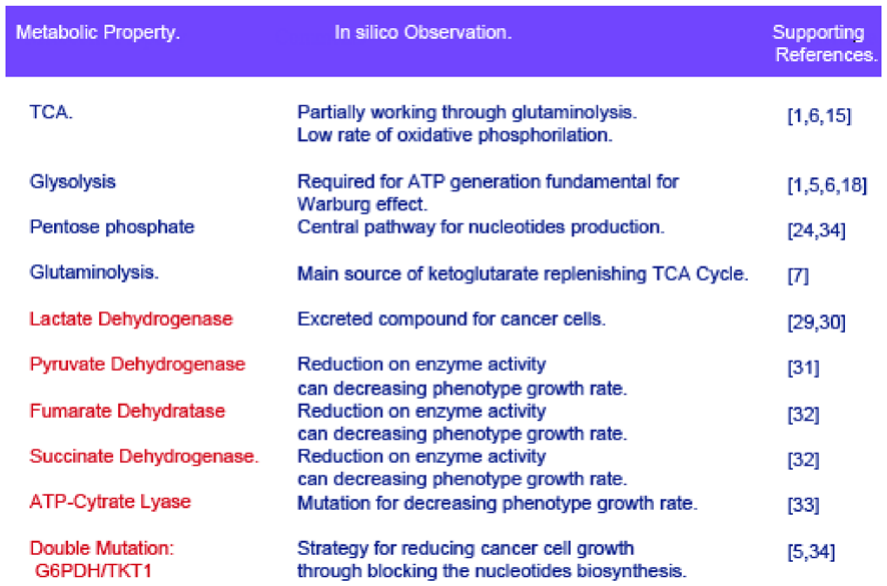
Physiological assessment of *in silico* interpretations. A proper assessment of *in silico* results is required to ensure proper reconstruction. Ten proofs were used here to evaluate the physiological consistency of the results from constraint-based modeling. In sequential order, columns indicate the metabolic property computationally analyzed, *in silico* predictions and its consistency with some representative references. The blue text in column 1 represents metabolic pathways, while red lines indicate the effects that enzyme mutation has on cancer cell growth.

All together, the complete set of benchmarks used for supporting this reconstruction are shown in *Figure 7*. In light of these results, our constraint-based modeling represents an effort to construct a computational platform that serves as a guide for accomplished descriptive and predictive analysis about cancer metabolisms, thereby creating a dialogue with an experimental counterpart.

### High throughput technology and cancer variability

Previous sections were devoted to constructing a metabolic network, simulating growth rate and evaluating its predictive scope based on published findings about cancer cells. From a conceptual view, metabolic activity resulting from our constraint-based analysis in nature represents an average behavior obtained from a cancer cell population whose variability and stochasticity on phenotype have been neglected. However, it is well known that the cancer cell population in tumors is heterogeneous, and a subsequent question is, then, to what extent cancer cell diversity in tumors can display heterogeneous metabolic activity [18]. A significant contribution to this issue was published regarding a cervical uterine cancer study. It concluded that significant variability within tumor regions mainly is concentrated on genes participating in transcriptional regulatory and metabolic mechanisms [18]. Thus, with the purpose of estimating the extent of the cellular heterogeneity inherent in tumors could affect the metabolic activity of our reconstruction, we have done a statistical analysis of more than 33 samples of expression profiling stored in the *GEO* repository at NCBI (accession *GSE5787*) obtained from several regions of 16 patients with cervical cancer [18]. In contrast to the methodology used by the original report [18], our variability analysis was quantified through classical statistics over the expression data obtained from *affymetrix* technology, *see methods section*. In agreement with the original findings that the expression of genes acting on signal transduction, regulatory mechanism and metabolic pathways was significantly varied, we obtained that genes participating in *oxidative* phosphorylation potentially induce a variability on enzymatic activity within the population, see Table S2 in supplementary material.

Guided by this result, we conclude that even though some genes involved in metabolism can be expressed quite variably in tumors, expression profiling reported for cervical cancer may suggest that most of the metabolic pathways included in this reconstruction are uniformly expressed at a population level, being the exception *oxidative phosphorylation*. Computational and experimental assessment for verifying this heterogeneity and exploring its biological consequences will be an issue to address in future.

## Discussion

Metabolic alterations constitute a hallmark in cancer cells: They are required for driving transformation, progression and spreading cancer in tissues [16]. Consequently, development of computational procedures for identifying those enzymes with an influential role on cancer cell growth constitutes an active line of research essential to establishing the bases of a rationalized design of drugs with the desired therapeutic effects [16]. In our study, a metabolic reconstruction integrating the best-known metabolic pathways participating in cancer development was accomplished: *glycolysis*, *pentose phosphate pathway*, *oxidative phosphorylation*, *glutaminolysis* and *TCA cycle*.

To assess the practical scope of the metabolic reconstruction, four criteria were evaluated: 1) the model's ability to simulate growth rate in *Hela* cells, 2) its ability to identify the global metabolic activity during cell proliferation; 3) the expected phenotype on growth rate when variations on enzymatic activity are induced; and 4) the effect that cancer heterogeneity has on metabolic reconstruction. Qualitative assessment of points 1 to 3, summarized by Figure 2 and 6, were successfully evaluated *in silico* and have led us to suggest that constraint-based modeling can be used as a descriptive and predictive framework to study the metabolic alterations supporting cancer in cells.

Although this computational study constitutes a good start in genome scale modeling in cancer metabolisms, our reconstruction and modeling is far from complete [36], and improvements, additional tests and more specific studies should be undertaken in future. Between the most relevant issues we name the follows:

1. It has been reported that the expression of *M2-PK*, a *specific-tumor pyruvate kinase*, may enhance cell biosynthetic capabilities by decreasing the transformation of *phosphoenolpyruvate (PEP)* to *pyruvate* and shunting the intermediary substrates upstream of *PEP* toward biosynthetic pathways required for cancer cell growth [1], [24]. This qualitative behavior was reproduced by constraint-based modeling only when a proper ratio among the objective function's components is tune by one that may quantify a more reliable conversion in cancer cell growth, *see* *Figure 6 D*. According with previous studies, an isoform of *pyruvate kinase PKM1*, *PKM2*, is highly represented during cancer cell growth such that it induces a reduced metabolic activity over pyruvate kinase producing a beneficial effects on cancer cell growth [27]. Consistently with this fact, our model can mirror this fact by identifying a region in phase plane whose decreased activity of *pyruvate kinase* can induce an increment on cancer growth rate, such as occurs with the switching between *PKM1* and *PKM2* during cell transformation. An inverse effect on cancer cell growth is observed at a threshold activity of *PKM1*, red line in Figure 6(D), a result that requires of additional experimental studies. This result makes evident the need of determining proper coefficients in the objective function's components to obtain meaningful biological interpretations, specially when exploring the finest of details in metabolic behavior.
2. In this study, we have selected a partial metabolic reconstruction in order to permit a comparison between *in silico* predictions and published findings in literature, such that, this approach facilitated a reliable assessment of the *in silico* results, *see* *Figure 7*. This primer modeling functions as a benchmarks that will allow us to sequentially introduce other pathways into the description. The reconstructed human metabolic network [36] constitutes an excellent source for extending our studies and potentially evaluate the role that new pathways have to sustain cancer phenotype.
3. From a genetic point of view, over expression of oncogenes and/or dysfunction of tumor repressors induce changes in genetic circuits that trigger the temporal transition between normal and cancer cell phenotype. Additional analysis will be required to explore how regulatory networks will constrain metabolic phenotype [37], [38] and to what extent the development of dynamic formalism can contribute to unraveling the main principles behind the robustness of cancer cells [39], [40]. In our opinion, both issues are relevant to distinguish the biological mechanism governing proliferation in cancer and normal functioning cell from a systems biology point of view.

## Methods

### Flux Balance Analysis

Constraint-based modeling is a computational frame for analyzing and exploring the phenotype space of metabolic networks obeying mass conservation and steady-state assumption [21], [22]. Thus, beginning from metabolic reconstruction of cancer cells, we applied linear programming to identify those metabolic fluxes (ν*_j_*, *j = 1,2…n*) that maximize biomass production when constrained by thermodynamics and mass balance principles;
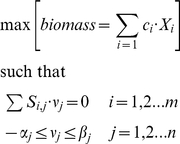
where *S_i,j_* represents the stoichiometric coefficient of metabolite *i* participating in the *j* reaction. In addition, biomass is represented by a linear combination of metabolites essential to cell growth (*X_i_*) and whose contribution to biomass production are weighted by coefficients (*c_i_*). Irreversible constraints and enzyme capacities for each metabolic reaction are quantified by parameters *α_j_* and *β_j_*.

### Dynamic Flux Balance Modeling

To model the temporal growth rate, we have assumed that along the time line, it is possible to identify a time scale, *Δt*, where the steady-state condition can be applicable. Briefly, assuming that at the moment of initiation, the cell environment has a glucose concentration, *s^o^_c_* and a cell density *X_0_*, the algorithm underlying dynamic constraint-based modeling consists of an iterative process among the following steps [21]. 1) Glucose concentrations *s_c_* available for the cell must be identified. 2) The glucose concentration then should be scaled to define quantity available per unit of biomass and per unit of time:
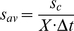
where *s_av_* denotes the initial concentration of glucose available in the environment. 3) Assuming a steady-state condition for metabolism, maximal growth rate, μ, at the interval of time Δ*t* is calculated through the linear programming algorithm [21], [23] and consequently growth rate and the new substrate glucose uptake rate *S_u_* are updated. 4) At the next described time step *(t+Δt)*, glucose concentration is obtained through the solution of the classic set of differential equations:
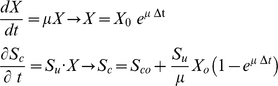
where *X* is the cell density, *S_u_* is the available concentration of glucose and μ represents the growth rate. In our simulation, the algorithm was repeated until null concentration of glucose was reached. The algorithm was implemented using *Cobra Toolbox* package in *Matlab* software [28].

### Flux Variability and Enzyme Essentiality analysis

*Flux Variability analysis* was applied to explore metabolic redundancy in optimal phenotype. Thus, this procedure identifies the range of numerical values for each flux over the reconstruction, while still satisfying the given constraints and optimizing a selected objective function [23]. *Enzyme essentiality analysis* was used to quantify to what extent the deletion of an enzymatic reaction affects the optimization of selected objective function. In order to identify those metabolic reactions obeying low *flux variability* and high *essentiality* we selected two threshold values. First, we defined as *essential* those metabolic reactions whose deletion reduced by 50% or more the original objective function. Second, reactions with low flux variability were those whose absolute values on *flux variability* were less or equal to 50% of the maximal flux variation along the entire distribution. Those reactions intersecting both sets constituted the target enzymes reported in Figures 3 and 4. Both computational analyses were accomplished in a *Matlab* environment and *COBRA toolbox*.

### Sampling null space of the stoichiometric matrix

The identification of metabolic fluxes (ν) obeying the steady-state condition, 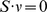,with ***S*** as the stoichiometric matrix, was accomplished by sampling the null space of *S* by an Artificial Center Hit and Run Algorithm (ACHR) [41], [42]. Basically, this algorithm defines an initial point along the null space of ***S***. Once this point is defined, the algorithm calculates “warm-up” points by an iterative procedure. These warm-up points are stored in a matrix **W** by which a centroid **x_c_** is calculated. Finally the sample points are calculated by selecting one point y_n_ in the **W** matrix and moving in the direction vector given by (**x_c_−y**). The new vector **y_n+1_** is substituted by the previous point **y_n_** in **W**. The centroid is recalculated, and this process continues iteratively until a desired number of sample points are reached. ACHR was done using the COBRA tool box [28]. Overall, 10,000 sampled points belonging to the null space of the stoichiometric matrix were included in the analysis.

### Comparative analysis between in silico and experimental growth kinetics

To verify that the kinetic growth curve obtained *in silico* can exhibit a comparable behavior with the experimentally obtained growth curve from *Hela* cell lines, we have applied a normalization procedure in both curves. We have denoted ***G*** as a cell growth matrix whose entry ***g*** *_i,j_* represents the *j* replicate of cellular density measured at time *i*, with *i = 1….5* (five days) and *j = 1…6* (six replicates per day). In addition, <***A***> represents the average growth vector whose entries ***A_i_*** indicate the average over rows of ***G***. The normalization applied on both curves, theoretical and experimental, was obtained through this transformation
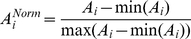
where *A_i_* represents the average cellular density at sample *i*, and *min* and *max* represent the minimal and maximal numerical value of the *i* row of <***A***>. In other words, above normalization permits selection of a framework in which the minimal value of ***A_i_^Norm^*** is equal to zero and a maximal value equal to a unit.

### Microarray analysis

In order to have an idea of how cancer heterogeneity can influence metabolic phenotype, we accomplished a statistical analysis of microarray data on 33 samples of cervical uterine cancer among 16 women patients. The analysis used previously published microarray data [18] stored in *GEO* (repository at *NCBI* under accession GSE5787) and analyzed in R with *affy* package in *bioconductors,* http://www.bioconductor.org/. To identify those genes that significantly vary with in the 33 samples, a *t-statistics* was accomplished selecting those genes with a *log-ratio* higher than 1.2 and a *p-value* less than *0.01*. The set of genes identified and its corresponding information (such as symbol, description, reference chromosome location, gene ontology, and participation in metabolic pathways) are shown in *Table S2*.

### Cell Culture Conditions and Growth Kinetics

Growth rate were obtained through standard technique of crystal violet procedure. The cell line HeLa was obtained from the Unidad de Diferenciacion Celular y Cáncer at FES-Zaragoza UNAM. Cancer cell line was cultured in RPMI-advanced 1640 serum-free media (Gibco BRL, USA) with red phenol and antibiotic-antimycotic solution (10,000 units penicillin, 10 mg streptomycin, and 25 µg amphotericin B per mL). The cells were incubated in 5% of CO2 and humidity saturation at 37°C. Cells were cultured in 1mL of RPMI-Advanced medium in 24-well cell culture plates (BD Falcon, USA) starting from an estimated cellular population of 10^5^ cells. Every day the medium for 6 different wells was removed and 100uL of glutaraldehyde at 1.1% were added, until you have enough samples to complete the kinetics. After this the glutaraldehyde is removed from each well and the plate is left at room temperature until dryness is reached. Violet crystal at 0.1% is added during 20 minutes and the wells are then washed with bidistillate water. The water is removed and then citric acid at 10% was added and shaken during 20 minutes. The absorbance at 600 nm was obtained for the supernadant in each well.

## Supporting Information

Table S1 Metabolic reconstruction for central metabolism in cancer.
(0.03 MB XLS)

Table S2 Genes with a significative variation on gene expression in cervical cancer.
(0.51 MB XLS)
